# Anti-Inflammatory and Antioxidant Properties of Plant Extracts

**DOI:** 10.3390/antiox10060921

**Published:** 2021-06-07

**Authors:** María Jesús Rodríguez-Yoldi

**Affiliations:** 1 Department of Pharmacology and Physiology, Veterinary Faculty, University of Zaragoza, 50013 Zaragoza, Spain; 2 CIBERobn, ISCIII, IIS Aragón, IA2, 50009 Zaragoza, Spain; mjrodyol@unizar.es

Since the ancient times, a great variety of plants have been used for therapeutic purposes. Most parts of plants have been used as extracts and may possess anti-inflammatory and antioxidant properties related to diseases such as diabetes, atherosclerosis, neurodegenerative, or cancer. In addition, plant extracts, as anti-inflammatory agents, can regulate the composition of the gut microbiota.

Fruits and vegetables contain a large amount of compound phytochemicals responsible for their medicinal properties, such as polyphenols, carotenoids, phytosterols, and polysaccharides. Currently, phytochemical and ethnobotanical studies are being carried out in order to identify the mechanism of action of a wide variety of natural compounds present in plant extracts. 

In this way, certain ailments whose etiology involves immune dysfunction or persistent inflammation can be protected by plants consumption by the downregulation of pro-inflammatory cytokines, COX, and reducing the translocation of NF-kB to the nucleus. Also, bioactive principles of plants can regulate the oxidative stress caused from an imbalance in the production of reactive oxygen species (ROS) and the antioxidant capability of the cell enzymes.

This Special Issue of *Antioxidants* is dedicated to current research on the aspects related to the anti-inflammatory and antioxidant properties of plant extracts, as well as the modulators and pathways involved in these therapeutic actions. In this issue, 16 original research papers and 6 reviews have been published.

In this context, it is known that polyphenols are the most abundant antioxidants in the human diet. They represent a variety of bioactive compounds that are capable of preventing and controlling diseases related to stress diseases. Thus, the review realized by Jung et al. provides an overview of the therapeutic benefits and targets of *Salvia miltiorrhiza* extracts, including inflammation, fibrosis, oxidative stress, and apoptosis [1]. However, it must be taken into account that the content of polyphenols in plants varies remarkably between species, geographical area, part of the plant tested, etc. In this sense, Bhatt and Debnath [2] studied, in blueberries (*Vaccinium* spp.), the genetic diversity of antioxidant properties in relation to the total phenolic and flavonoid content, and associated with molecular markers. 

Phytosterols belong to the triterpene family and are structurally similar to cholesterol. They are known for their cholesterol-lowering effects, anti-inflammatory and antioxidant properties, and the benefits they offer to the immune system. The review by Vezza et al. provides an overview of these bioactive compounds and their therapeutic potential in the fields of obesity and metabolic disorders, in relation to oxidative stress, inflammatory status, and gut dysbiosis [3].

As indicated, plant extracts have been used as natural therapeutic agents against inflammation, characterized by an overproduction of inflammatory mediators such as ROS and pro-inflammatory cytokines. In this sense, studies in murine macrophage cells (RAW264.7), with extracts from *Notopterygium incisum,* revealed an anti-inflammatory effect by interfering with lipopolysaccharide (LPS) binding to Toll-like receptor 4 (TLR4), signaling and activating the antioxidative nuclear factor erythroid 2-related factor 2 (Nrt2). In addition, this factor increased the expression of the antioxidant protein heme oxygenase-1 (HO-1), superoxidase dismutase (SOD) and catalase (CAT) [4]. Similar results were obtained with different organ extracts of *Abeliophyllum distichum* by suppressing ROS production and/or inhibiting the activation of protein kinases (ERK1/2) when treating RAW264.7 cells with LPS [5]. Likewise, in LPS-activated RAW2647.7 cells, sorghum extracts inhibited the production of nitric oxide (NO), interleukin-6 (IL-6), and ROS, with ethanolic extracts showing greater anti-inflammatory activity related to tannin content [6]. Berries are a rich source of phytochemicals, especially phenolics, which are well known for their protective activity against many chronic diseases. In addition, berries also contain a complex mixture of volatile compounds that are responsible for its aroma. Gu et al. [7] found that both berry phenols and volatile fractions show an anti-inflammatory effect on LPS-stimulated RAW264.7 cells through the suppression of the nuclear factor-kappa B (NF-kB) signaling pathway.

The inflammation of the colon is a highly currently prevalent disease. Kopiasz et al. found that oat beta-glucans exert an antioxidant effect in animals with colitis induced by 2, 4, 6-trinitrobenzene sulfonic acid (TNBS), with greater effectiveness in removing the systemic effects of colon inflammation [8].

Increased levels of ROS and a low-grade chronic inflammation in multiple organs have been demonstrated in obesity. A comparative study of the antioxidant and anti-inflammatory effects of leaf extracts from four different *Morus alba L.* genotypes *(Filipina, Valenciana Temprana, Kokuso, and Italia),* in mice with diet-induced obesity, was performed by Leyva-Jimenez et al. [9]. The results showed that *Filipina* and *Italia* methanolic extracts have the higher antioxidant and anti-inflammatory effect due to the presence of compounds such as protocatechuic acid or quercetin-3-glucoside, and they could be developed as a complementary treatment for obesity and metabolic disorders. Continuing with studies related to inflammation diseases, Mainka et al. determined the antioxidant activity and composition of extracts from eleven species of plants traditionally used in inflammatory skin diseases in Poland [10].

Likewise, a review by Noh et al. showed that several natural products possess antioxidant properties and androgenic activities on productive factors and hormones, and therefore the management of infertility with these products could be taken into account [11].

Regarding to the powerful antioxidant activity of polyphenols, these compounds can help prevent or treat several diseases related to oxidative stress such as cancer, diabetes, neurodegenerative, autoimmune, cardiovascular, and ophthalmic diseases, among others [12]. However, plant extracts can also be used to alleviate the harmful side effects of drugs. Thus, a study by Abd El-Rahman et al. [13] showed that *Saussurea lappa* has a remarkable protective effect against triamcinolone acetonide (glucocorticoid extensively used) via its anti-inflammatory, anti-apoptotic, and antioxidant capacity. In addition, the plant extract can be a viable alternative to the serious problem of microbial resistance to antibiotics. In this way, natural extracts from *Pinus pinaster* bark, rich in phenols, showed potent antibacterial activity against Gram-positive bacteria [14]. Likewise, a review by Bogdan et al. shows the antimicrobial activity of *Vitis vinifera* by-products and their application in oral care [15].

The antioxidant capacity of polyphenols can also be used as an anti-aging potential, as is shown in a study carried out by Moliner et al. [16] with rosemary flowers (*Rosmarinus officinalis* L.), in *Caenorhabditis elegams*.

A large number of studies have suggested that the consumption of polyphenols could have beneficial effects on the central nervous system, by improving its blood flow as well as preventing or delaying the onset of neurodegenerative disorders [12]. In this way, peripheral neuropathy (PN) is a clinical problem that affects many patients and with few effective therapies. Recently, the interest in natural dietary compounds in human health has led to a great deal of research, especially in PN. Curcumin is a polyphenol extracted from the root of *Curcuma longa*, and has been used in Asian medicine for its anti-inflammatory, antibacterial, and antioxidant properties. In a review by Caillaud et al., in vivo and in vitro studies on this molecule in the treatment of different PN have been shown, highlighting its molecular mechanisms of action [17].

Dietary intervention could play a key role in both the prevention and treatment of diabetes mellitus. Pancreatic beta cells are vulnerable to oxidative stress, which causes beta cell death and dysfunction in diabetes mellitus. In this sense, *Broussonetia kazinoki* Siebold was used to prevent or treat beta cell damage in diabetes [18]. Likewise, *Pinus pinaster* bark extracts showed hyperglycemic activity determined by α-amylase and α-glucosidase assays [14].

Cancer is one of the leading causes of death in the world. Cancer treatments, based on chemotherapy, surgery, radiotherapy, etc., are currently applied. However, sequela of such cancer therapies and cachexia are a problem to the patients, hence the interest in applying natural compounds in the treatment of this disease. A review by Lee et al. focuses on the potential of plant extracts as great therapeutic agents in controlling oxidative stress and inflammation associated with tumoral environment. Thus, this review shows how some antioxidants derived from plants inhibited the proliferation of cancer cells and inflammation after surgery, and others prevented the apoptosis of healthy cells through the elevation of antioxidant enzymes or anti-apoptotic proteins and controlling cytokine levels [19]. Therefore, dietary polyphenols might play a dual role in cancer treatment, since they have been proved to be beneficial for chemoprevention as well as for cancer treatment. Thus, grape stem extracts showed an antiproliferative effect in colon (Caco-2) and breast (MCF-7 and MDA-MB-231) cell lines through apoptosis cell death associated with a modification of the mitochondrial potential and ROS levels. Additionally, grape stem extracts showed an antioxidant effect on non-cancerous intestinal cells that could protect the intestine from diseases related to oxidative stress [20]. Likewise, *Capsicum annuum* L., incorporated into liposomes, reduced the ROS in the human hepatoma (HepG2) cell line, and the extracts promoted the expression of endogenous antioxidants, such as catalase, superoxide dismutase, and glutathione peroxidase through the Nrf-2 pathway [21]. *Pinus pinaster* bark extracts also showed an anticancer effect by decreasing cell viability in human lung cancer cells [14]. Studies realized with *Satureja hortensis L.* herb have shown that the budding phase alcohol extracts of this plant contain the largest amounts of polyphenols, including rutin and rosemary acid, which promote the radical scavenging activity and antioxidant properties with an anticancer effect on tumor skin cells [22]. In this way, *Phoenix dactylifera* seed extract can also be used as a skin whitening agent by attenuating melanogenesis in B16F10 cells by downregulating protein kinase A (PKA) signaling pathways [23].

Taken together, the papers included in this Special Issue illustrate examples of recent advances in the treatment and prevention of disease with plant extracts. In some work it has been shown that fruit and vegetable residues (waste by-products), rich in bioactive compounds, can also be used for therapeutic purposes. In addition, in some papers, the alternative application of natural compounds in the case of resistance to antibiotics, or in harmful side drug effects, has been shown. The general objective was to compile research and review articles that cover the latest trends in the area, and thus benefit researchers and readers in advancing their knowledge on the subject.
